# Incidence of emerging multidrug-resistant organisms and its impact on the outcome in the pediatric intensive care

**DOI:** 10.1186/s43054-021-00071-1

**Published:** 2021-11-01

**Authors:** Ahmed R. Rezk, Somaia Abdelhammed Bawady, Nashwa Naguib Omar

**Affiliations:** 1 Department of Pediatrics, Faculty of Medicine, Ain Shams University, Cairo, Egypt; 2 Department of Clinical Pathology, Faculty of Medicine, Ain Shams University, Cairo, Egypt

**Keywords:** Multidrug-resistant organisms (MDROs), Pediatric ICU, Antimicrobial resistance

## Abstract

**Background:**

Healthcare-associated infections (HCAI) are a worldwide threat in intensive care units particularly in the pediatric intensive care units with a major cause of morbidity and mortality among this age group. The aim of the study is to determine the prevalence and risk factors predisposing to multidrug-resistant organisms (MDROs) infections among pediatric intensive care unit (PICU) patients at Ain Shams Pediatric University Hospitals as well as determining mortality and morbidity rates along with the length of stay at PICU.

**Results:**

Culture results revealed that of the 282 patients evaluated, only 26 (9.2%) were MDROs (half of the affected patients had Acinetobacter species (50%) and the rest of them were free, 256 (90.8%). Our study revealed that the majority of MDROs were isolated from sputum in more than half of the patients 19/32 (59.3%) followed by whole blood in 10/32 (31.2%) and urine in 3/32 (9.4%). Pulmonary system was the most common affected site and was mainly colonized by MDR Acinetobacter (71.4%) followed by MDR Klebsiella (41.6%). Regarding MDR risk factors in our PICU, multivariate logistic regression analyses showed significant relationship between MDROs and age under 1 year (odds ratio [OR] 2.4554; 95% confidence interval [95% CI] (1.072–5.625); *p* = 0.043) and underlying pulmonary disease (OR 2.417; 95% CI (1.014–5.761); *p* = 0.592). A statistically significant higher mortality was detected in patients colonized with MDROs 9/26 (34.6%) versus MDROs non-colonized patients 32/256 (12.5%) [*P*=0.002]. Moreover, MDROs infection has negative significant risk with discharged patients ([OR] 0.269; [95% CI] (0.111–0.656); *p* = 0.002). Additionally, patients infected with MDROs did have significantly greater PICU stay than those non-infected [median (IQR), 16.5 (10.7–22), 5 (4–8), *P*=0.00] and have longer ventilation [median (IQR), 15.5 (10–18), 3 (2–10), *P*=0.00].

**Conclusion:**

Prevalence of MDROs (9.2%) was low among PICU cases at Ain Shams University Hospitals. Most common MDROs were Acinetobacter and Klebsiella followed by pseudomonas species. The frequency of gram-negative organisms is much more common than gram-positive organisms. An increasing rate of antimicrobial resistance with increasing mortality and morbidity among PICU patients is observed worldwide; even for new categories, so, strict infection control programs should be implemented.

## Background

Healthcare-associated infections (HCAI) are a global major threat in intensive care units. Infections affecting patients in Pediatric and Neonatal intensive care units (PICU and NICU) range from 6 to 12% and 10 to 25% respectively [1].

Multidrug-resistant organisms (MDROs) are one of the most serious challenges in healthcare-associated and community-acquired infections [2], primarily due to gram-negative bacteria (GNB) which are increasing worldwide with higher mortality and morbidity than gram-positive bacteria [3].

Since the discovery of penicillin in 1928, antibiotics transformed the management of bacterial infections and saved millions of lives. Many years later, an increased spread of antimicrobial-resistant microorganisms made bacterial infections to become a threat again. This antimicrobial-resistance phenomenon carries a very heavy burden on healthcare, with 23,000 and 25,000 estimated annual deaths respectively in the USA and in Europe. Moreover, some further studies expected a rising impact on global health through the years, leading to more than 10 million annual deaths worldwide in 2050 [3].

The antimicrobial resistance is rapidly increasing in regions with poor hygiene and uncontrolled use of antimicrobials [4]. The epidemiology of MDR organisms differs across different countries and regions [5] being higher in the developing countries versus the developed ones (23.5%, and 6.1%, respectively) [6].

Patients in intensive care units (ICUs) are a major target population for hospital infectious pathogens. Due to their immune-compromised condition, broad-spectrum antibiotic usage, and medical manipulations that disrupt the natural defenses of the host including the use of invasive devices [e.g. intravascular devices, intubation, nasogastric tubes, and urinary catheters (UC)] [6].

The mortality and morbidity rates are noticeably higher in those infections due to extensive antimicrobial resistance and the critical state of the patients. Therefore, monitoring ICU infectious microorganisms and recording their antimicrobial resistance are of great importance to ensure the prompt organization of measures related to preventive, control, and therapeutic actions [7].

Therefore, the aim of this study was to determine the prevalence and risk factors predisposing to MDROs infections among PICU patients at Ain Shams Pediatric University Hospital as well as determining mortality rates and morbidities along with length of stay at PICU.

## Methods

The present study was a prospective study conducted on 282 pediatric patients recruited from pediatric intensive care unit of Children’s Hospital at (faculty of medicine, Ain Shams University Hospitals, Egypt) Cairo, Egypt. This study was approved by the hospital's institutional review board and the ethics committee of Ain Shams University. Informed consent was taken from parents of each child enrolled in the study within duration of 6 months; from June 2019 to January 2020. Demographic data were collected along with admission diagnosis, fate, length of hospital stay and number of ventilation days.

Patients involved in this study were all patients admitted to the PICU during the period of the study, aged more than 1 month and less than 18 years. Patient’s variables were recorded by full history and complete physical examination. Recorded parameters were: Vital data: temperature, respiratory rate, heart rate and blood pressure. Full cardiac, respiratory, abdominal and neurological examination was done.

The selected cases were further subdivided into 2 groups regarding the presence of MDR organisms, cases with MDR organisms and non-MDR organism’s cases (on the basis of the antibiotic susceptibility patterns).

Laboratory and radiological investigations done were: Complete blood count : Two ml of fresh venous blood was collected in tube containing EDTA as an anticoagulant, complete blood counts were performed using sysmex XT-1800i (Symex, Kobe, Japan). C-reactive protein : was measured using the semi-quantitative latex agglutination test (Avitex CRPkit, Omega Dignostic Limited, Scotland, UK). Serum electrolytes measured were: Na+, K+, BUN, Creatinine and ALT. Blood samples for serum biomarkers were withdrawn on admission and repeated when signs of infections were clinically detected. Additionally, Blood, sputum, and urine samples were collected from each patient to perform culture and antimicrobial sensitivity.

### Microbiologic methods and MDR definition

Standard microbiological methods were used for the isolate identification and antimicrobial susceptibility testing from all collected samples and were performed in the Central Microbiology Laboratories, Faculty of Medicine (Ain shams University Hospitals), Egypt.

### MDR

According to Centre for Disease Control & Prevention (CDC), MDR is defined as acquired non-susceptibility to at least one agent in three or more antimicrobial categories.

### Specimens collection

Blood cultures, sputum, and urine samples were obtained from all cases under complete aseptic conditions. Samples were collected on admission and after 72 h after admission.

### Processing of Specimens

BACTEC blood culture vials BACTEC BD (Becton, Dickinson and company (continuous monitoring blood culture system)), once admitted to the clinical microbiology laboratory, were directly incubated aerobically at 35±1 °C into the BACTEC fluorescent series instrument. All positively signaled blood vials, urine, and sputum samples were inoculated on blood agar and Maconkey agar. All plates were incubated at 35±1 °C overnight. Blood and sputum samples were additionally inoculated on chocolate agar plates and were incubated in 5–10% CO_2_ jar overnight at 35±1 °C.

### Analytical methods

Verifying the identity of the recovered isolates was carried out by various standard conventional microbiological methods including; colony macroscopic morphology, microscopic examination, and other biochemical tests. Further identification was performed using an automated identification system (Vitek® 2 automated system (Biomerieux, France).

Antimicrobial susceptibility testing of all isolates was performed by both Kirby-Bauer disk diffusion method and MIC determination using Vitek® 2 automated system (Biomerieux, France), according to the Clinical and Laboratory Standards Institute (CLSI) 2015 guidelines and interpretative criteria [8].

Used antibiotic classes, penicillins, tetracyclines, cephalosporins, quinolones, carbapenems, lincomycins, macrolides, sulfonamides, aminoglycosides, glycopeptide antibiotics, and oxazolidinones, were tested against gram-positive and gram-negative isolates: ampicillin/sulbactam (10/10 μg), cefoxitin (30 μg), ceftazidime (30 μg), cefpodoxime (30 μg), cefotaxime (30 μg), ceftriaxone (30 μg), imipenem (10 μg), meropenem (10 μg), amikacin (30 μg), gentamicin (10 μg), tobramicin (10 μg), ciprofloxacin (5 μg), levofloxacin (5 μg), tetracycline (30 μg), doxycycline (30 μg), piperacillintazobactam (100/10 μg), oxacillin (1 μg), vancomycin (30 μg), erythromycin (15 μg), and norfloxacin (10 μg); all disks were from Oxoid, UK.

Methicillin resistance in all staphylococcus species was detected using the cefoxitin disk test (30 μg; Oxoid, UK), for prediction of MecA gene-mediated methicillin resistance in the staphylococcus species, as recommended by the Clinical and Laboratory Standards Institute [8].

Gram-negative bacilli isolated were considered multidrug-resistant (MDR) organisms when they show resistance to three or more antimicrobial classes [8].

### Statistical analysis

IBM SPSS statistics (V. 26.0, IBM Corp., USA, 2019) was used for data analysis. Data were expressed as median and percentiles for quantitative non-parametric measures in addition to both number and percentage for categorized data.

The following tests were done:

1. Comparison between two independent groups for non-parametric data using Wilcoxon rank sum test.
2. Chi-square test for comparison between 2 independent groups as regards the categorized data.
3. Calculated relative risk assessments (relative risk ratio or RRR) that measure how many times the risk was present among diseased individuals as that among non-diseased ones. They were calculated as absolute figures and as a standard error of estimate (95P).

The probability of error at 0.05 was considered sig., while at 0.01 and 0.001 are highly significant.

## Results

Two hundred eighty-two of pediatric ICU patients were enrolled in the study; their demographic and clinical characteristics are shown in Table 1. The median (IQR) of age was 1.5 (0.5–3.1), nearly half of them were males (54.3%). The most common underlying cause for pediatric ICU admission was post-operative (42.2%) followed by chest (19.5%) and cardiac diseases (10.6%). Only (31.5%) were ventilated and majority of them were discharged (85.5%).

**Table 1:** Descriptive statistics of demographic and clinical data of all participants

Parameters	Patients (*n*=282) *n* (%)
Age	
Median (IQR)	**1.5 (0.5–3.1)**
Sex	
F M	**129 (45.7%)** **153 (54.3%)**
Number of Admission days	
Median (IQR)	**6 (4–10)**
Number of ventilation days (*n*=89)	
Median (IQR)	**6 (2–15)**
Fate	
Discharged Died	**241 (85.5%)** **41 (14.5%)**
MDR	
Positive Negative	**26 (9.2%)** **256 (90.8%)**
Diagnosis	
Cardiac Chest CNS GIT Hematological Metabolic Renal Septic shock Surgical	**30 (10.6%)** **55 (19.5%)** **22 (7.8%)** **9 (3.2%)** **11 (3.9%)** **16 (5.7%)** **9 (3.2%)** **11 (3.9%)** **119 (42.2%)**

Infection with MDR bacteria regarding species, affected samples, and antibiotic combinations used for treatments are shown in Table 2. Of the 282 patients evaluated, only 26 (9.2%) were colonized with MDR gram-negative bacteria (half of the affected patients had Acinetobacter species (50%) and the rest of them were free 256 (90.8%). Primarily, sputum samples were most affected 19 (59.3%) mainly by MDR Acinetobacter (71.4%) and MDR Klebsiella (41.6%). Piperacillin Tazobactam + Colistimethate sodium combination was the main combination used in (50%) of cases followed by Imipenem+ Colistimethate sodium (34.6%).

**Table 2:** Descriptive statistics of affected systems, MDR microorganisms and antibiotic combinations used according to the University's Antimicrobial stewardship program

Parameter	*n* (%)
System affected (*n*=32)*	
Sputum	**19 (59.3%)**
Blood	**10 (31.2%)**
Urine	**3 (9.4%)**
Prevalence of MDR microorganisms (*n*=28)**	
Acinetobacter MDR	**14 (50%)**
Klebsiella MDR	**12 (42.8%)**
Pseudomonas MDR	**2 (7.1%)**
Antibiotic combinations used (n=26)	
Piperacillin Tazobactam + Colistimethate sodium	**13 (50%)**
Imipenem+ Colistimethate sodium	**9 (34.6%)**
Ceftazidime-Clindamycin –Amikacin	**1 (3.8%)**
Colistimethate sodium + Ciprofloxacin	**1 (3.8%)**
Piperacillin + Amikacin	**1 (3.8%)**
Meropenem+ Amikacin	**1 (3.8%)**

Descriptive statistics of each MDR micro- organism regarding antibiotic combinations and affected systems are shown in Table 3. Piperacillin Tazobactam + Colistimethate sodium combination was used in 5 out of 14 (35.7%) of Acinetobacter sp., in 8 out of 12 (66.6%) of Klebsiella sp., and in 2 out of 2 (100%) of Pseudomonas sp. Moreover, Imipenem+ Colistimethate sodium combination was used in 6 out of 14 (42.8%) of Acinetobacter sp. and in 4 out of 12 (33.3%) of Klebsiella sp. With regards to the recovery of microorganisms from different samples, 10 out of the 14 (71.4%) Acinetobacter sp. were recovered from sputum samples, whereas 5 out of the 12 (41.6%) of Klebsiella sp. were recovered from those samples.

**Table 3:** Descriptive statistics of each MDR microorganism regarding antibiotic combinations and affected systems

Parameter	MDR Acinetobacter (*n*=14)	MDR Klebsiella (*n*=12)	MDR Pseudomonas (*n*=2)
Antibiotic used			
Piperacillin Tazobactam + Colistimethate sodium	**5 (35.7%)**	**8 (66.6%)**	**2 (100%)**
Imipenem+ Colistimethate sodium	**6 (42.8%)**	**4 (33.3%)**	**-**
Ceftazidime-Clindamycin – Amikacin	**-**	**1 (8.3%)**	**-**
Colistimethate sodium and Ciprofloxacin	**1 (7.1%)**	**-**	**-**
Piperacillin Amikacin	**1 (7.1%)**	**-**	**-**
Meropenem+ Amikacin	**1 (7.1%)**	**-**	**-**
Samples			
Sputum	**10 (71.4%)**	**5 (41.6%)**	**-**
Blood	**2 (14.3%)**	**4 (33.3%)**	**1 (50%)**
Urine	**2 (14.3%)**	**3 (25%)**	**1 (50%)**

All Acinetobacter species were resistant to ampicillin/sulbactam, cefoxitin, ceftazidime, cefpodoxime, cefotaxime, ceftriaxone, imipenem, meropenem, amikacin, gentamicin, tobramicin, ciprofloxacin, levofloxacin, and norfloxacin. However, 60% of them were resistant to tetracycline and doxycycline. All Klebsiella species were resistant to ampicillin/sulbactam, iperacillintazobactam, cefoxitin, ceftazidime, cefpodoxime, cefotaxime, ceftriaxone, imipenem, meropenem, amikacin, gentamicin, tobramicin, ciprofloxacin, levofloxacin, tetracycline and doxycycline, and norfloxacin. All pseudomonas species were resistant to cefoxitin, ceftazidime, cefpodoxime, cefotaxime, ceftriaxone, imipenem, meropenem, amikacin, gentamicin, tobramicin, ciprofloxacin, levofloxacin, and norfloxacin. However, 70% of them were resistant to piperacillintazobactam, and 80% were resistant to tetracycline and doxycycline.

Staphylococcus coagulase-negative isolates represented only 5 out of 282 samples collected (1.77%) and *Staphylococcus aureus* represented 4 out of 282 samples (1.41%); all were sensitive to cefoxitin and non-MRSA strains. Additionally, *Echerichia coli* isolated represented only 2 out of the total of 282 (0.7%) samples collected and were non-MDR strains.

The risk factors for MDR pathogen colonization in 26 patients compared with 256 patients without this colonization are shown in Table 4. The risk factors identified [OR; 95% CI] included age < 1 year [2.4554; 1.072–5.625] and underlying chest disease [2.417; 1.014–5.761]. In our study, colonization with MDR pathogens tends to prolong the ventilation and admission days and is associated with a bad prognosis.

**Table 4:** Descriptive and comparative statistics between patients infected with MDR microorganisms and patients without infection

Parameter	Patients with MDR (*n*=26)	Patients without MDR (*n*=256)	*Z*,X*	*P*	Odds ratio 95CI (lower-upper)
Age					
Age (< 1 year)	**16 (61.5%)**	**101 (39.5%)**			**2.4554** **(1.072–5.625)** **For age < 1 year** **Pos. Sig. risk**
Age (1–6 years)	**6 (23.1%)**	**124 (48.4%)**			
Age (7–18 years)	**4 (15.4%)**	**31 (12.1%)**			
Sex					
F	**13 (50%)**	**116 (45.3%)**	0.209*	**0.648**	
M	**13 (50%)**	**140 (54.7%)**			
Number of Admission days					
Median (IQR)	**16.5 (10.7–22)**	**5 (4–8)**	**− 5.212**	**0.0**	
Number of ventilation days (*n*=89)	**(*n*=20)**	**(*n*=69)**			
Median (IQR)	**15.5 (10–18)**	**3 (2–10)**	**− 3.96**	**0.0**	
Fate					
Discharged	**17 (65.4%)**	**224 (87.5%)**	9.291*	**0.002**	**0.269** **(0.111–0.656)** **For discharged** **Neg. Sig. risk**
Died	**9 (34.6%)**	**32 (12.5%)**			
Diagnosis					
Cardiac	**2 (7.6%)**	**28** (**10.9%)**			**2.417** **(1.014–5.761)** **For chest** **Pos. Sig. risk**
Chest	**9 (34.6%)**	**46(17.9%)**			
CNS	**0 (0.0%)**	**22 (8.6%)**			
GIT	**1 (3.8%)**	**8 (3.1%)**			
Hematological	**1 (3.8%)**	**10 (3.9%)**			
Metabolic	**2(7.7%)**	**14 (5.5%)**			
Renal	**1 (3.8%)**	**8 (3.1%)**			
Septic shock	**1 (3.8%)**	**10 (3.9%)**			
Surgical	**9 (34.6%)**	**110 (42.9%)**			

Overall colonization showed an increasing trend over time. Specimens were collected on admission as baseline samples followed by sample 1 (after 72 h). Only 4 out of 26 patients were colonized with MDR bacteria on admission. Only 6/26 (23%) patients were colonized with MDR bacteria in more than one site, only 2/26 (7.7%) patients have been infected with more than 2 organisms.

N.B. 26 patients are infected with MDR microorganisms

Systems affected (*n*=32)* as many systems are affected per patient

MDR microorganisms (*n*=28)** as 2 or 3 MDR microorganisms can infect one patient

*Z*: Wilcoxan rank sum test

X*: Chi-square test

## Discussion

MDR organisms represent a worldwide threat in ICU hospitalized children; they affect disease infection control and are accompanied with high mortality rates. Treatment cost is correspondingly increased secondary to the prevalence of resistant pathogens requiring more expensive therapies [2]

The prevalence of infections sustained by MDR bacteria in ICU patients varies in different regions of the world. In North America, a study on critically ill patients with pneumonia (DEFINE study) reported a 14.1% rate of MDR infections, while a large study on nosocomial bloodstream infections conducted in 24 ICUs distributed worldwide (EUROBACT study) showed on average a 47.8% MDR rate, including 20.5% and 0.5% of isolated microorganisms with extensively drug-resistant (XDR) and pan-drug-resistant (PDR) patterns, respectively, with major variations between different countries ranging from 8% (Australia) to more than 75–80% (Turkey, Greece, Croatia, Serbia) [9, 10].

In the present study, the incidence of colonization of MDR bacteria among all admitted patients was 26/282 (9.2%) which was comparable to the incidence reported by a study done in three PICUs of one tertiary children’s hospital in Italy 8.72% (79/906) [11], which is significantly lower than that of older reports with incidence of MDROs that ranged from10 to 25% in PICU [12, 13].

Our study conducted on 282 PICU patients, the culture results revealed 68 isolates only 26 (38.2%) were MDROs and the rest 42(61.8%) were non-MDROs. This rate was lower than that reported even in other regions in Egypt as reported in a study done in 2 pediatric ICUs in Pediatric Hospital-Cairo University [14] 98/106 (92.45%) and in Neonatal and Pediatric Intensive Care Units of Beni-Suef University Hospital145/169 (85.8%) [2]. Moreover, our rate was lower than the reported prevalence in King Chulalongkorn Memorial Hospital-Thailand 30/58 (52%) [15]. This major difference can be explained by variations in the sample size, different demographic regions, and inadequate implementation of infection control measures [2].

Possible explanations for the high percentage of MDROs in some PICUs can be extrapolated by abuse of antibiotics in the outpatient settings, treating viral infections with antibiotics, inappropriate dose, and incomplete course of antibiotics [2]. It has been stated that the university/teaching hospitals that usually operate as referral hospitals generally report higher infection rates [16].

In a review of several studies, Cosgrove showed that there is an association between the development of antimicrobial resistance in some microorganisms and the increase in mortality rates, the duration of hospital stay, and the cost of health care. Inadequate therapy or delay in therapy and the presence of underlying disease were thought to be responsible for the adverse outcomes associated with antimicrobial-resistant infections [17].

Similarly, a statistically significant higher mortality was detected in our patients colonized with MDROs 9/26 (34.6%) versus MDROs non-colonized patients 32/256 (12.5%) [*P*=0.002]. Moreover, MDROs infection has negative significant risk with discharged patients ([OR] 0.269; [95% CI] (0.111–0.656); *p* = 0.002).

Additionally, patients infected with MDROs did have significantly greater PICU stay than those non-infected [median (IQR), 16.5 (10.7–22), 5 (4–8), *P*=0.00] and have longer ventilation [median (IQR), 15.5 (10–18), 3 (2–10), *P*=0.00].

Our results matching the previous studies, which have reported that the patients with multi-drug resistant infections (MDRI) were hospitalized and treated in the ICU for considerably longer stay and had lower survival rates compared to other patient groups [11].

In the present study, the mortality rate was 41/282(14.5%) which was matching that reported in a study done in Beni-Suef university hospital (Egypt) 10/80 (12.5%) [2]. However, higher rates were detected in other developing countries ranged between 10–53.6%. In contrast, very low mortality rates were reported in the developed countries [2].

In our study the incidence of HAI 68/282 (24.1%) which is comparable to pediatric ICUs in Pediatric Hospital-Cairo University106/378 (28%) [16 ]. In Europe, incidence of HAI ranges from 1% in general pediatric wards to 23.6% in PICUs [18], while a nation point prevalence study of PICU in the USA found the incidence to be 11.9% [19].

A study on the Extended Prevalence of Infection in Intensive Care (EPIC) II revealed that 51% of patients were considered to be infected while in ICU. Infections were of respiratory origin in 64% of cases. The most frequent isolated microorganism was *Staphylococcus aureus* (20.5%). However, the overall predominance was for Gram-negative organisms as a group: 62.2% (Klebsiella spp., Pseudomonas spp., E. coli, Enterobacter spp., and Acinetobacter spp.) [20].

This is in harmony with this study as we detected only MDR gram-negative bacteria (MDR-GNB), MDR Acinetobacter was isolated in half of the cases colonized with MDR strains followed by MDR Klebsiella 12/28 (42.8%), and MDR Pseudomonas was isolated from only 2/28 (7.1%) patients. Like our study, predominance of MDR-GNB was also reported by some studies such as those done in Italy [21], Tunisia[ 22], and the Philippines [22].

Over the years, and as a result of the increasing lack of effective antimicrobial agents against resistant gram-negative microorganisms, MDR gram-positive microorganisms have been much less than MDR gram-negative strains [23].

Nowadays effective treatment against MDR bacteria is few or missing for specific PDR strains. Beta-lactam antibiotics were considered the first-line treatment against many microorganisms and this was due to their high safety profile and broad efficacy, for many decades. However, bacterial production of β lactamase enzymes progressively increased globally making beta-lactams ineffective as first-line treatments for nosocomial infections in many countries of the world. In the last years, the use of Carbapenems as first-line empiric treatments in many critically ill patients grew rapidly leading to a noticeable increase in the incidence of Carbapenem-resistant bacteria, by various mechanisms of resistance [24].

In our PICU, Piperacillin Tazobactam and Colistimethate sodium combination was effective in 13/26 (50%) while Imipenem and Colistimethate sodium was effective in 9/26 (34.6%).

Acinetobacter baumannii (AB) is a frequent cause of nosocomial acquired infection in critically ill patients [24]. In fact, it is considered to be the third microorganism that is responsible for ventilator-associated pneumonia in the European ICU patients, after *S. aureus* and *P. aeruginosa* [25]. Although Carbapenems are usually considered the first-line agents for the treatment of severe infections caused by AB, their usage is becoming limited in many areas because of the increasing resistance [23]

These data matching our study as MDR Acinetobacter is responsible for half 14/28 (50%) of the MDROs isolations mainly causing pulmonary infections in the majority of cases 10/32 (71.4%) and was responding to antibiotic combinations primarily to carbapenems (Imipenem) and Colistimethate sodium in 6/26 (42.8%) of patients and to a lesser extent to Piperacillin Tazobactam and Colistimethate sodium combination in 5 (35.7%) patients.

In many European regions, Klebsiella pneumoniae producing Carbapenemase is considered as one of the most common MDR gram-negative microorganisms in critically ill patients [26, 27]..

In this study, MDR Klebsiella pneumoniae was the second most common organism after MDR Acinetobacter baumannii isolated from 12/28 (42.8%) and foremost sensitive to Piperacillin Tazobactam and Colistimethate sodium combination in 8/12 (66.6%) and causing both pulmonary infection in 5/12 (41.6%) and sepsis 4/12 (33.3%).

Pseudomonas aeruginosa (PA) is one of the commonest causes of health-care-associated infections and is responsible for severe bloodstream, pulmonary, urinary tract, and soft tissue infections in ICU cases [28]. On the contrary, MDR Pseudomonas aeruginosa was the least organism isolated in our study in only 2/28 (7.1%) patients.

Our study revealed that the majority of MDROs were isolated from sputum in more than half of the patients 19/32 (59.3%) followed by whole blood in 10/32 (31.2%) and finally urine in 3/32 (9.4%). Conversely, Wang et al. showed that out of the 79 cases of MDRIs, 43 (54.4%) cases were detected in the whole blood, 23 (29.1%) cases in sputum [11], matching several previous studies which detected most of MDR isolates at blood cultures (69.7%) [2]. Similar findings were obtained in other studies in Egypt [29–31] and other different countries (including China, Mexico, South Africa, and Kenya) [32–34].

Identifying risk factors for infection development caused by multidrug-resistant bacteria can help health care providers prevent nosocomial infections. This is much more important when we consider the slow development of new effective anti-microbial agents and the increasing prevalence of MDRI, especially in the PICU [11].

Regarding risk factors for acquiring nosocomial infections due to resistant organisms in our PICU, multivariate logistic regression analyses showed significant relationships between MDROs and age under 1 year (odds ratio [OR] 2.4554; 95% confidence interval [95% CI] (1.072–5.625); *p* = 0.043) and underlying pulmonary diseases (OR 2.417; 95% CI (1.014–5.761); *p* = 0.592).

These risk factors were also reported by another study, which concluded that acquiring nosocomial infections due to resistant organisms in PICU patients is more likely in patients with transplants and those with underlying lung disease [17].

Similarly, age under 2 years along with the length of hospital stay (more than 3 days) was identified as risk factors for acquisition of MDR during hospitalization in PICU in Tunisia [ 22]. Young age as a risk factor might reflect an inherent risk to acquire MDR by environmental contamination [ 22].

However, other risk factors were detected by several studies such as Atta et al. [35], who found that hematologic diseases and healthcare-associated infection had a significant relationship with colonization of multi-drug-resistant gram-negative bacteria (MDR-GNB). In another study in PICU in Italy which concluded the length of PICU stay, the duration of mechanical ventilation > 5 days, parenteral nutrition, coma, urinary catheter indwelling, snd invasive operation, two or more antibiotics use were associated with MDROs [11].

## Conclusion

Prevalence of MDROs (9.2%) was low among PICU cases at Ain Shams University Hospitals. Most common MDROs were Acinetobacter and Klebsiella followed by pseudomonas species. The frequency of gram-negative organisms is much more common than gram-positive organisms. An increasing rate of antimicrobial resistance with increasing mortality and morbidity among PICU patients is observed worldwide; even for new categories, so, strict infection control programs should be implemented.

## Abbreviations

HCAI: Healthcare-associated infections
PICU and NICU: Pediatric and Neonatal intensive care units
MDROs: Multidrug-resistant organisms
GNB: Gram-negative bacteria
ICUs: Patients in intensive care units
UC: Urinary catheters
CDC: Centre for Disease Control & Prevention
CLSI: Clinical and Laboratory Standards Institute
XDR: Extensively drug-resistant
PDR: Pan-drug-resistant
EPIC: Extended Prevalence of Infection in Intensive Care
